# Reliability and validity of the Chinese version of the autoimmune bullous disease quality of life (ABQOL) questionnaire

**DOI:** 10.1186/s12955-017-0594-z

**Published:** 2017-02-02

**Authors:** Baoqi Yang, Guo Chen, Qing Yang, Xiaoxiao Yan, Zhaoxia Zhang, Dédée F. Murrell, Furen Zhang

**Affiliations:** 1 Institute of Dermatology and Department of Dermatology, The First Affiliated Hospital, Anhui Medical University, 81 Meishan Road, Hefei, Anhui 230032 China; 2 Department of Dermatology, Shandong Provincial Institute of Dermatology and Venereology, Shandong Provincial Academy of Medical Science, 27397 Jingshi Road, Jinan, Shandong 250022 China; 3 Department of Dermatology, Shandong Provincial Hospital for Skin Diseases, 27397 Jingshi Road, Jinan, Shandong 250022 China; 4 Department of Dermatology, Laiwu Institute of Dermatology, 76 South Huayuan Road, Laiwu, Shandong 271100 China; 5 Department of Dermatology, St George Hospital, University of New South Wales, Gray Street, Kogarah, Sydney, NSW 2217 Australia

**Keywords:** Chinese, Autoimmune bullous disease, Quality of life, Reliability, Validity, Questionnaire

## Abstract

**Background:**

The autoimmune bullous diseases quality of life (ABQOL) questionnaire was recently developed by an Australian group and has been validated in Australian and North American patient cohorts. It is a 17-item, multidimensional, self-administered English questionnaire. The study aimed to validate the Chinese version of the ABQOL questionnaire and evaluate the reliability in Chinese patients.

**Methods:**

The Chinese version of the ABQOL questionnaire was produced by forward-backward translation and cross-cultural adaptation of the original English version. The ABQOL questionnaire was then distributed to a total of 101 patients with autoimmune bullous diseases (AIBDs) together with the Dermatology Life Quality Index (DLQI) and the 36-item Short Form Health Survey (SF-36). Validity was analyzed across a range of indices and reliability was assessed using internal consistency and test-retest methods.

**Results:**

The Chinese version of the ABQOL questionnaire has a high internal consistency (Cronbach’s alpha coefficient, 0.88) and test-retest reliability (the intraclass correlation coefficient, 0.87). Face and content validity were satisfactory. Convergent validity testing showed that the correlation coefficients for the ABQOL and DLQI was 0.77 and for the ABQOL and SF-36 was −0.62. In terms of discriminant validity, there was no significant difference between the proportions of insensitive items in ABQOL and DLQI (*p* = 0.236). There was no significant difference between the proportions of insensitive items in ABQOL and SF-36 (*p* = 0.823).

**Conclusions:**

The Chinese version of the ABQOL questionnaire has adequate validity and reliability. It may constitute a useful instrument to measure disease burden in Chinese patients with AIBDs.

**Electronic supplementary material:**

The online version of this article (doi:10.1186/s12955-017-0594-z) contains supplementary material, which is available to authorized users.

## Background

Autoimmune bullous diseases (AIBDs) are a group of disorders characterized by autoantibodies directed against structural proteins present in the desmosome and hemidesmosome of the skin and mucosal membranes. The AIBDs include various forms of pemphigus, bullous pemphigoid (BP), epidermolysis bullosa acquisita (EBA), linear IgA bullous dermatosis (LABD), dermatitis herpetiformis (DH), and pemphigoid gestationis (PG) [1]. Blistering lesions have a large effect on the quality of life (QOL) [2]. Treatment can also affect the QOL of patients with AIBDs. For example, the steroids and immunosuppressive agents used to control AIBDs may cause serious adverse effects. Therapy-related complications significantly contribute to the mortality in patients with AIBDs [3]. It is thus of great importance to pay considerable attention to the patients’ QOL and psychological states as well as clinical status.

Currently, the 36-Item Short Form Health Survey (SF-36) and the Dermatology Life Quality Index (DLQI) are the most reported measures to evaluate the QOL of AIBD patients. The Chinese versions of the DLQI and SF-36 have been validated in China [4, 5]. These generic and dermatology-specific QOL instruments show that patients with AIBDs have a significant decrease in QOL compared with the greater population [6, 7]. Recently, a disease-specific instrument, the AIBDQOL (ABQOL) questionnaire was developed by an Australian group [8]. It is a 17-item, multidimensional, self-administered English questionnaire. Validation in Australian patients showed that the ABQOL had a moderate correlation with scores on the DLQI and the General Health subscale of the SF-36 in terms of convergent validity. In terms of discriminant validity, the ABQOL was found to be more sensitive than the DLQI [8].

The ABQOL was found to be a reliable patient-reported outcome measure in both Australian and North American patient cohorts with satisfactory internal consistency (Cronbach’s alpha coefficient, 0.84–0.90) and test-retest reliability (intra-class correlation coefficient, 0.92–0.93) [8, 9]. As the first validated instrument with the specificity of the questions maximizing the ability to detect any changes in disease process, ABQOL questionnaire is a promising disease specific outcome measure to assess the QOL of patients with AIBDs [9]. In this study, we aimed to establish and validate a Chinese version of the ABQOL questionnaire and evaluate its reliability in Chinese-speaking patients with AIBDs.

## Methods

### Participants

AIBDs patients were recruited from the Department of Dermatology of Shandong Provincial Hospital for Skin Diseases. Patient inclusion criteria were: at least 18 years of age, a confirmed diagnosis of an AIBD, and sufficient education to complete the questionnaire without assistance. All patients with AIBDs were diagnosed according to clinical manifestations, histopathological findings, direct and indirect immunofluorescence assay. Indirect immunofluorescence utilized monkey esophagus as substrates. Pemphigus was also verified by ELISA for desmogleins 1 and 3. BP and PG were verified by BP180-NC16a ELISA and BP230 ELISA, respectively. EBA was verified by indirect immunofluorescence assay performed on sodium chloride-split skin and ELISA for type VII collagen. Paraneoplastic pemphigus (PNP) was verified by a history tumor and polymorphous mucocutaneous eruption besides immunofluorescence assay.

### Forward-backward translation of the ABQOL into Chinese

Permission was obtained from the original English version of ABQOL copyright holder. The English ABQOL was translated into Chinese by a translation company with the relevant linguistic background. The Chinese version of the ABQOL was then independently back translated into English by another certified language service provider with no access to the original English questionnaire. The back-translated version was then reviewed against the originals by the original authors of the ABQOL and minor revisions were made. Five dermatologists reviewed the Chinese translation and reached consensus.

To pilot test and refine the questionnaire, we recruited 10 AIBDs patients to complete the questionnaire. An experienced interviewer systematically pre-tested patients by asking them what they thought the question was asking, what the answers were, and to explain how they decided their answers. Based on the feedback from the 10 patients, we revised several words in the questionnaire to adapt to the language and culture in China. No points of misunderstanding were detected. Subsequently, the final Chinese version of the ABQOL questionnaire was administered for the study. The English and Chinese versions of the ABQOL questionnaire are presented in Additional file 1.

### Formal questionnaire

A single investigator administered the survey to reduce investigator bias. All patients (76 outpatients and 25 inpatients) completed the Chinese version of the ABQOL questionnaire without assistance on day 0. The DLQI and the SF-36 were also completed on day 0 to evaluate their correlation with the ABQOL. The outpatients were also asked to take home a stamped addressed envelope containing the ABQOL questionnaire to complete on day 7 and return to the investigator. The inpatients completed the questionnaires on day 7 in the hospital. The general patient characteristics, including disease stage, time for completion of the questionnaire, number of school years completed, profession, and gender were also recorded.

### Reliability

Internal consistency was measured using Cronbach’s alpha coefficient, which also tested for construct validity [7]. Test-retest reliability was assessed by comparing the total ABQOL scores in a subset of patients who completed the questionnaire on both day 0 and day 7. To reduce the recall bias, a 7-day interval was selected for the retest. The intra-class correlation coefficient (ICC) was calculated to determine the ability of the tool to give concordant results at different times.

### Validity

Validation of the Chinese version of ABQOL included the assessment of face, content, construct, convergent, and discriminant validity [6]. Face and content validity were established by forward-backward translation of the ABQOL and by review of the questionnaire by the panel of bullous disease experts. Convergent validity was determined by correlating the ABQOL scores with the scores obtained from DLQI or SF-36. Discriminant validity was assessed by comparing the proportions of insensitive items in the ABQOL and DLQI (or ABQOL and SF-36) [8, 10].

### Factor analysis

Construct validity was also assessed through factor analysis [8]. Exploratory factor analysis and principal component analysis followed by Oblimin rotation with Kaiser normalization were performed to assess the dimensionality of the 17 items and to validate the structure of the Chinese version of the ABQOL questionnaire. Significance was defined as a loading greater than 0.4 or less than −0.4.

### Statistical analysis

All statistical analyses were performed using SPSS v10.01 software (SPSS, Inc., Chicago, IL). A p value of <0.05 was considered statistically significant.

## Results

### Demographics

**Fig. 1:**
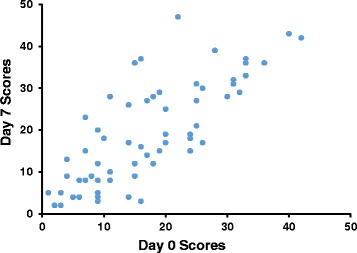
The test-retest reliability of the ABQOL (*n* = 61)

A total of 101 patients with AIBDs were recruited between March 2015 and April 2016. All patients completed the day 0 questionnaire and a subset of 61 patients (60.4%) completed the day 7 questionnaire. All the participants declared that there was no obstacle in understanding the items. Patient demographics are presented in Table 1. Of the 101 patients recruited, 67 were men and 34 were women. Patient ages ranged from 18 to 77 years, with a mean age of 50.02 years. The number of school years completed ranged from 4 to 18 years, with a mean of 9.37 years. The mean time for completion of the ABQOL questionnaire was 5.64 min (range 1.37–15.00 min). Most of the patients had pemphigus vulgaris (PV, *n* = 57), followed by BP (*n* = 25), pemphigus foliaceus (PF, *n* = 11), DH (*n* = 3), LABD (*n* = 2), EBA (*n* = 1), PG (*n* = 1) and PNP (*n* = 1). The patient with PNP had a history of thymoma resection. The mean (standard deviation, SD) ABQOL scores for patients with PV was 17.23 (1.35); for BP, 16.60 (2.09); and for the remaining patients, 17.16 (2.09) (Fig. 1). The mean disease duration of all patients was 17.82 months (range 10 days to 108 months). The clinical disease stages were defined as baseline (*n* = 5), complete remission during tapering (*n* = 23), complete remission on minimal therapy (*n* = 29), complete remission off therapy (*n* = 2), control of disease activity (*n* = 30), partial remission on minimal therapy (*n* = 1), time to control of disease activity (*n* = 8), and relapse/flare (*n* = 2) [11].

**Table 1:** Demographic characteristics of the patient cohort

Variable	Value
Age (years), mean (range)	50.02 (18–77)
Course of disease (months), mean (range)	17.82 (0.33–108)
Schooling years (years), (range)	9.37 (4–18)
Completion time (min), mean (range)	5.64 (1.37–15)
Patients enrolled, n (%)	
Outpatients	76 (75.25)
Inpatients	25 (24.75)
Sex, n (%)	
Male	67 (66.34)
Female	34 (33.66)
Autoimmune bullous disease, n (%)	
Pemphigus vulgaris	57 (56.44)
Bullous pemphigoid	25 (24.75)
Pemphigus foliaceus	11 (10.89)
Dermatitis herpetiformis	3 (2.97)
Linear IgA bullous dermatoses	2 (1.98)
Eidermolysis bullosa acquisita	1 (0.99)
Paraneoplastic pemphigus	1 (0.99)
Pemphigoid gestationis	1 (0.99)
Clinical stages, n (%)	
baseline	5 (4.95)
complete remission during tapering	23 (22.77)
complete remission on minimal therapy	29 (28.71)
complete remission off therapy	2 (1.98)
control of disease activity	30 (29.70)
partial remission on minimal therapy	2 (1.98)
time to control of disease activity	8 (7.92)
relapse/flare	2 (1.98)

### Reliability and validity

The internal consistency and construct validity of the Chinese version of ABQOL were acceptable, with a Cronbach’s alpha of 0.88 (Table 2). The test-retest reliability of the questionnaire was also acceptable, with an ICC value of 0.87 (Table 2, Fig. 1). With regard to convergent validity, the correlation coefficient for the Chinese ABQOL and DLQI was 0.77 (*p* < 0.001), indicating a moderate correlation between the ABQOL and DLQI. The correlation coefficient for the Chinese ABQOL and SF-36 was −0.62 (*p* < 0.001). There was no significant difference in the proportion of insensitive items between ABQOL and DLQI (Fisher exact test, *p* = 0.236), or between ABQOL and SF-36 (the Pearson Chi-square test, *p* = 0.823). Statistical analyses of validity and reliability measures are presented in Table 2.

**Table 2:** Validity and reliability for the ABQOL

	Statistic	Result for 17 items questionnaire
Face and content validity	Forward-backward translation, culture-adaption and review of the bullous experts panel	satisfactory
Convergent validity	Correlation with DLQI	*r* = 0.77, *p* < 0.001
	Correlation with SF-36	*r* = −0.62, *p* < 0.001
Discriminant validity	DLQI	*p* = 0.236
	SF-36	*p* = 0.823
Internal consistency	Cronbach’s alpha	alpha = 0.88
Test-retest reliability	ICC	*r* = 0.87

*Abbreviations: ABQOL* Autoimmune Bullous Disease Quality of Life Questionnaire, *DLQI* Dermatology Life Quality Index, *SF-36* Medical Outcome Study 36-item short-form questionnaire, *ICC* intraclass correlation coefficient

### Factor analysis

The Kaiser-Meyer-Olkin measure of sampling adequacy (0.804) and the Bartlett test of sphericity (*p* < 0.001) suggested that factor analysis of the data was appropriate [12]. The Catell scree plot suggested that three factors (symptom, mucosal, and psychosocial) should be retained, representing 53.51% of the cumulative variance. The rotation matrix obtained by the exploratory principal components analysis followed by Oblimin rotation [12] indicated that nine items loaded on symptom subscale (questions 1, 2, 3, 4, 5 and 9, 10, 11, 12); two items loaded on mucosal subscale (questions 6 and 7); and six items loaded on psychosocial subscale (questions 8, 13, 14, 15, 16, 17). The three dimensions represented are shown in Table 3.

**Table 3:** The principal component and factor analysis of the Chinese version of ABQOL

Question No.	Description of item	Factor 1 symptoms	Factor 2 mucosal	Factor 3 psychosocial
1	pain	**.796**	.038	-.162
2	itch	**.765**	-.228	-.096
3	clothing changes	**.521**	.212	-.030
4	healing	**.664**	.026	.042
5	bathing or showering	**.736**	-.044	.089
6	pain (mouth)	.027	**.817**	.034
7	gingival bleeding	.091	**.711**	-.002
8	food avoidance	.053	.270	**.360**
9	embarrassment	**.473**	.136	.395
10	depression	**.582**	.278	.124
11	anxiety	**.524**	.270	.151
12	family/friends	**.494**	-.321	.406
13	sexual activity	.263	.077	**.571**
14	relationships	-.023	-.395	**.844**
15	social life	.325	.253	**.487**
16	work and study	.112	.417	**.506**
17	discrimination	-.170	.254	**.616**

Extraction method: principal components analysis; rotation method: Oblimin with Kaiser normalization; Bold items load on the assigned factor

## Discussion

In the present study, we established a Chinese version of the ABQOL questionnaire and performed reliability and validity tests of this questionnaire in a Chinese cohort of AIBDs patients. Our results showed a little higher internal consistency with a Cronbach’s alpha of 0.88 than the value validated in the Australian cohort (0.84) where the original English ABQOL was developed [8]. The English ABQOL has also been validated in a North American patient cohort with satisfactory internal consistency (Cronbach’s alpha coefficient, 0.90). It is known that a Cronbach’s alpha of above 0.70 would be ideal to evaluate the reliability of patient-reported measures for internal consistency of a questionnaire [13]. Thus, this version of Chinese ABQOL questionnaire will be a reliable patient-reported outcome measure for evaluating the QOL of Chinese AIBDs patients. In addition to the reliability, a Cronbach’s alpha of 0.88 also indicates that this Chinese version of ABQOL has satisfactory construct validity. The test-retest reliability coefficient of our questionnaire was 0.87, close to the values in the original study and in the North American cohort (intra-class correlation coefficient, 0.92 and 0.93, respectively). This data suggest that the Chinese ABQOL questionnaire will yield consistent results under similar conditions.

We noticed that more men were recruited in this study. This gender bias would attribute to that AIBDs usually affects the elderly and the differential educational levels between the men and women aged people in China. Most of the older adults in China, especially women, are poorly educated. In contrast, ABQOL is a self-reported questionnaire. Many elder Chinese women AIBDs patients could not completed the questionnaire by themselves and thus, these patients were excluded. Most of the patients were in the category of PV (*n* = 57, 56.4%) and BP accounted for 24.8% (25 of 101), with the remaining in other categories. The ABQOL scores for patients with PV was 17.23 ± 1.35; for BP, 16.60 ± 2.90; and for the remaining patients, 17.16 ± 2.09. In contrast, the Australian cohort in the validation of the original ABQOL composed of 40% PV (28 of 70), 35.7% BP, and 24.3% of others. The mean (SD) ABQOL scores for patients in the three categories were 11.5 (5.5), 8.4 (5.5), and 11.9 (8.9), respectively. The North American cohort enrolled 39 patients, composed of 46.1% PV (*n* = 18), 28.2% BP (*n* = 11), and 25.6% of others. The ABQOL scores for PV and BP were 16.4 ± 2.9 and 10.8 ± 2.5, respectively. Thus, PV is more prevalent in our cohort and these patients had a higher mean ABQOL scores than those in the original validation study of the ABQOL while similar to those in the North American cohort. In contrast, the BP patients in our study had higher ABQOL scores than those in the other two studies. These differences might contribute to the different internal consistency of the questionnaire in the three cohorts.

Before the launching of the newly developed ABQOL for AIBDs patients, generic and dermatology-specific QOL instruments were utilized to monitor disease activity and to evaluate the effectiveness of care. The Medical Outcomes Study SF-36 and the dermatology-specific DLQI have acknowledged the significant decrease in QOL of patients with AIBDs compared with the greater population. Paradisi et al. found that patients with pemphigus had a markedly impaired overall QOL compared with healthy subjects [14]. There was a significant association between disease severity and lower SF-36 scores in these patients. High prevalence of psychiatric comorbidity was also observed in pemphigus patients [15]. In addition to the SF-36, DLQI and General Health Questionnaires have also been used to monitor the QOL and psychological status of patients with pemphigus vulgaris [7, 16, 17].

Patients with symptom of itching and severe skin involvement had worse QOL and there was a negative correlation between DLQI score and duration of the disease [7]. Pemphigus patients were also recorded to have the psychiatric morbidity rates at 40% by GHQ-12 and 26% by ICD-10 [16, 17]. Besides pemphigus, the QOL of other AIBDs patients including BP and DH were also severely impaired as assessed by DLQI [18]. The newly developed ABQOL, however, was found to be more sensitive than the DLQI in determining the disease activity (*p* < 0.02) in the initial evaluation [8]. This ABQOL explores greater depth in a corresponding domain than that in a generic measure, enabling a heightened responsiveness to any changes that may occur. We revealed moderate to good convergence between the Chinese version of ABQOL and DLQI instruments (convergent validity, *r* = 0.77), even slightly higher than that found in the original study (0.64). The correlation coefficient (−0.62, *p* < 0.001) between the ABQOL and SF-36 was also higher than that observed in the original study (0.51) [8].

Similar as the original ABQOL study, the 17 items of the questionnaire were also classified as factors of three categories: psychological, physical, and social factors. However, there is an interesting difference in the categories of the item ‘food avoidance’ (question 8) between the Chinese version and the original ABQOL. In the original development of the ABQOL in Australia, this item was classified as a physical factor, meaning the hardness of the food may destroy the mucous membrane of the esophagus of the AIBDs patients. However, due to the Chinese traditional culture, food avoidance in the Chinese version of ABQOL is rather a behavior method for avoidant of certain food.

The original literature had validated that the disease-specific QOL instruments are more sensitive to changes in clinical status than general QOL measures [10]. However, in our study, there were 7, 7, and 18 insensitive items in the ABQOL, DLQI, and SF-36, respectively. The ABQOL was not more sensitive than SF-36 (*p* = 0.823) and DLQI (*p* = 0.236). The cultural difference and the poor schooling years were the likely reasons. For example, the word ‘holiday’ is an unfamiliar concept to many Chinese patients, especially to the rural patients. However, it is hard to find a more suitable word to replace it, we thus still use the original word ‘holiday’.

There are several limitations to this study. First, as the patients were recruited from a single hospital there might be patient selection bias. Second, the correlation between ABQOL and Autoimmune Bullous Skin Disorder Intensity Score (ABSIS), pemphigus disease area index (PDAI) or Bullous Pemphigoid Disease Area Index (BPDAI) [19] was not included in the study. A recently study illustrated that BPDAI was correlated with ABQOL [20].

## Conclusion

Our study findings indicate that the Chinese version of the ABQOL questionnaire has adequate validity and reliability. It can be used to monitor the disease activity of Chinese patients with AIBDs.

## Additional file

Additional file 1: ABQOL questionnaire. (DOC 68 kb)

## Abbreviations

ABQOL: Autoimmune bullous disease quality of life questionnaire
AIBDs: Autoimmune bullous diseases
BP: Bullous pemphigoid
DH: Dermatitis herpetiformis
DLQI: Dermatology life quality index
EBA: Epidermolysis bullosa acquisita
ICC: Correlation coefficient
LABD: Linear IgA bullous dermatosis
PG: Pemphigoid gestationis
QOL: Quality of life
SF-36: The 36-item short form health survey
