# Supplementary material for: Reliability and validity of the Chinese version of the autoimmune bullous disease quality of life (ABQOL) questionnaire
Source: Health Qual Life Outcomes. 2017 Feb 2;15:31. doi: 10.1186/s12955-017-0594-z (PMC5290597; doi:10.1186/s12955-017-0594-z)
Supplement: Additional file 1: — ABQOL questionnaire. (DOC 68 kb) [file 12955_2017_594_MOESM1_ESM.doc]

# ***ABQOL Questionnaire***

Name: __________________________________ Date:________________

DOB: _____________ Sex: M/F Contact Number:__________________

Pemphigus Subtype: Pemphigus Vulgaris Epidermolysis Bullosa Acquisita Bullous Pemphigoid Linear IgA Bullous Dermatoses

Pemphigus Foliaceus Mucous Membrane Pemphigoid

Other....................................................................................................

The following questions ask about the ways in which *blistering disease* affects your quality of life.

**Please choose an option from the right hand column which most closely correlates to how you felt *within the last week*.**

Please indicate the time started the survey: ______________ AM/PM

| 1. In regards to your blistering disease, does your skin burn, sting or hurt in any way? | - - All the time   - Sometimes   - Occasionally   - Never |
| --- | --- |
| 1. In regards to your blistering disease, does your skin itch? | - - All the time   - Sometimes   - Occasionally   - Never |
| 1. Have you had to change your clothing because of your blistering disease? | - - I have to be very careful with how tight my clothing is and what materials they are made of – I have had to change what I wear all the time   - I have had to change most of the things I wear   - I have had to change some of the things I wear   - I have never had to change what I wear |
| 1. Do you notice your skin heals slowly? | - - I notice this all the time   - I notice this sometimes   - I notice this occasionally   - I have never had this problem |
| 1. Do you have difficulty bathing or showering because of your blistering disease? | - - All the time   - Sometimes   - Occasionally   - Never |
| 1. In regards to your blistering disease, does your mouth have erosions which are painful? | - - All the time   - Sometimes   - Occasionally   - Never |
| 1. In regards to your blistering disease, do your gums bleed easily? | - - All the time   - Sometimes   - Occasionally   - Never |
| 1. Does your blistering disease result in you having to avoid food or drinks that you enjoy? | - - I can no longer eat any of the foods I used to enjoy   - I can eat some of the foods I enjoy   - I can eat most of the foods I enjoy   - I can eat anything I like |
| 1. As a result of your blistering disease, are you embarrassed about your appearance? | - - All the time   - Sometimes   - Occasionally   - Never |
| 1. Do you feel depressed or angry because of your blistering disease? | - - All the time   - Sometimes   - Occasionally   - Never |
| 1. Do you feel anxious or cannot relax as a result of your blistering disease? | - - All the time   - Sometimes   - Occasionally   - Never |
| 1. Do you worry that friends and family find your blistering skin condition tiresome? | - - All the time   - Sometimes   - Occasionally   - Never |
| 1. Is your blistering disease causing sexual difficulties? | - - All the time   - Sometimes   - Occasionally   - Never |
| 1. Does your blistering disease affect relationships with friends or loved ones? | - - I have had to end a relationship because of my disease OR I cannot have a relationship because of my disease   - Relationships are very difficult   - Relationships are a little difficult   - This has not affected my relationships |
| 1. Does your blistering disease affect your social life? | - - I cannot go out to socialize any more   - I can only go to some social events   - I can go to most social events   - My social life is not affected |
| 1. Does your blistering disease affect your work or study? | - - Yes, I can no longer work or study   - Yes, I find it difficult to work or study   - Yes, it is a little harder than before to work or study   - No, I am not affected **OR** this is not applicable |
| 1. Do employers discriminate against you because of your blistering disease? | - - I cannot find a job due to my blistering disease   - I have had to change jobs due to my blistering disease   - I still have my job but it is more difficult than before   - My employers are completely understanding **OR** this is not applicable |

Please indicate the time finished the survey: ______________ AM/PM

**Thank you for taking the time to complete this questionnaire**

**自身免疫性大疱病生活质量调查问卷**

姓名： 性别： 男/女 出生日期： _______________ 联系电话：________________

床号 住院号______________ 填写日期：_____________ 调查者：______________

疾病亚型：

□ 寻常型天疱疮 □ 获得性大疱性表皮松解症

□ 大疱性类天疱疮 □ 线状IgA大疱性皮病

□ 落叶型天疱疮 □ 黏膜性类天疱疮

□ 其他：___________

以下问题询问的是关于大疱病在哪些方面影响了您的生活质量。请根据最近**一周**来您的真实感受，在右侧一栏的选项中勾选出您认为最接近的一项。

**请记录填写该调查问卷的开始时间**： 时 分（上午/下午）

| 1. 关于您的大疱病，您的皮肤有烧灼感、刺痛感或是任何形式的疼痛吗？ | ○总是  ○有时  ○偶尔  ○从不 |
| --- | --- |
| 2. 关于您的大疱病，您感到皮肤瘙痒了吗？ | ○总是  ○有时  ○偶尔  ○从不 |
| 3. 由于患有大疱病，您不得不经常更换衣服吗？ | ○我不得不留心衣服有多紧身，衣物是用什么材料做的-我不得不总是更换我的衣服。  ○我需要更换大部分的衣服  ○我需要更换部分衣服  ○我从不需要更换我的衣服 |
| 4. 您注意到您的皮肤愈合的慢了吗？ | ○我总是注意到  ○我有时候能注意到  ○我偶尔注意到  ○我从未有这个问题 |
| 5. 由于患有大疱病，您感到洗澡或淋浴有困难吗? | ○总是  ○有时  ○偶尔  ○从不 |
| 6. 关于您的大疱病，您的口腔内有疼痛性的糜烂面吗？ | ○总是  ○有时  ○偶尔  ○从不 |
| 7. 关于您的大疱病，您的牙龈容易出血吗？ | ○总是  ○有时  ○偶尔  ○从不 |
| 8. 您的大疱病有没有使您不得不放弃一些您喜欢的食物或饮料？ | ○我已经不能再吃以前我喜欢的食物了  ○我能吃一部分我喜欢的食物  ○我能吃大部分我喜欢的食物  ○我能吃任何喜欢的食物 |
| 9. 由于患有大疱病，您会因自己的外貌感到尴尬吗？ | ○总是  ○有时  ○偶尔  ○从不 |
| 10. 由于患有大疱病，您感觉到郁闷或生气了吗？ | ○总是  ○有时  ○偶尔  ○从不 |
| 11. 由于患有大疱病，您感觉到焦虑不安或精神不能放松了吗？ | ○总是  ○有时  ○偶尔  ○从不 |
| 12. 您担心您的朋友和家人发现您患有大疱病而讨厌您吗？ | ○总是  ○有时  ○偶尔  ○从不 |
| 1. 由于患有大疱病，您的性生活感到困难了吗？ | ○总是  ○有时  ○偶尔  ○从不 |
| 14. 由于患有大疱病，您和朋友或爱人之间的关系受影响了吗？ | ○因为我的疾病，我不得不结束与朋友或爱人的关系。或者不能有朋友或爱人  ○与朋友或爱人的关系变得非常困难  ○与朋友或爱人的关系变得有一点困难  ○这并没有影响到我与朋友或爱人的关系 |
| 15. 您的大疱病影响到您的社交活动了吗？ | ○我再也不能出去参加社交活动了  ○我只能参加一部分社交活动  ○我能参加大多数的社交活动  ○我的社交活动未受影响 |
| 16. 您的大疱病影响到您的工作或学习了吗？ | ○是的，我不能再继续工作或学习了  ○是的，我觉得工作或学习困难了  ○是的，我的工作和学习比起以前来有点困难了  ○没有，我未受影响或该问题不适用 |
| 17. 由于患有大疱病，您受到老板的歧视了吗？ | ○由于我的大疱病我找不到工作  ○由于我的大疱病我不得不换工作  ○我仍然在原工作岗位但是工作起来比以前困难  ○我的工作伙伴对我表示完全理解或该问题不适用 |

请记录填写完成该调查问卷的时间： 时 分（上午/下午）**。**

感谢您抽出时间完成这个调查问卷。
